# NSUN4‐dependent m5C modification facilitates mitochondrial fission to accelerate diabetic nephropathy process via regulating SMURF1/CAMK1 axis

**DOI:** 10.1002/ctm2.70783

**Published:** 2026-08-27

**Authors:** Danyi Yang, Yinyin Chen, Hao Li, Guoyong Liu, Yang Gao, Liyu He

**Affiliations:** ^1^ Department of Nephrology The Second Xiangya Hospital of Central South University, Hunan Key Laboratory of Kidney Disease and Blood Purification Changsha City China; ^2^ National Clinical Research Center for Metabolic Diseases The Second Xiangya Hospital Central South University Changsha City China; ^3^ Department of Nephrology Hunan Provincial People's Hospital, The First Affiliated Hospital of Hunan Normal University Changsha City China; ^4^ Department of Nephrology The First Affiliated Hospital of Changde Vocational Technical College Changde City China

**Keywords:** CAMK1, diabetic nephropathy, m5C modification, mitochondrial, NSUN4, SMURF1

## Abstract

**Background:**

NOP2/Sun RNA methyltransferase family member 4 (NSUN4)‐mediated 5‐methylcytosine (m5C) modification has been implicated in diabetes‐related diseases. However, its role and molecular mechanism in diabetic nephropathy (DN) remain unclear.

**Methods:**

DN mouse model was constructed by injecting with streptozotocin (STZ). High glucose (HG)‐induced HK‐2 cells were used to establish a cellular model of DN. The levels of NSUN4, SMAD ubiquitination regulatory factor 1 (SMURF1), calcium/calmodulin‐dependent protein kinase 1 (CAMK1), and mitochondrial‐related proteins were analyzed using qRT‐PCR, western blot, or immunohistochemical staining. Renal injury in mice was evaluated using relevant kits and histological staining. Cell apoptosis, ROS production and proliferation were examined by TUNEL, dihydroethidium, MitoSOX Red and EdU staining. The interaction between SMURF1 and Aly/REF export factor (ALYREF) or CAMK1 was confirmed by RNA immunoprecipitation, Co‐IP and ubiquitination assay.

**Results:**

Upregulation of NSUN4 increased the m5C level in DN. Knockout of NSUN4 alleviated STZ‐induced renal injury in mice by repressing renal tubule cell mitochondrial fission and promoting mitochondrial fusion. NSUN4 downregulation reduced mitochondrial fission and promoted mitochondrial fusion to relieve HG‐induced HK‐2 cell apoptosis. NSUN4‐mediated m5C modification promoted SMURF1 mRNA stability by regulating ALYREF. SMURF1 overexpression rescued the suppressive effect of NSUN4 knockdown on cell mitochondrial fission and apoptosis. Besides, SMURF1 facilitated the ubiquitination and degradation of CAMK1. Furthermore, sh‐CAMK1 abolished the inhibitory effect of sh‐SMURF1 on cell mitochondrial fission and apoptosis. Meanwhile, downregulation of NSUN4 relieved STZ‐induced renal injury in DN mice by reducing mitochondrial fission via the SMURF1/CAMK1 axis.

**Conclusion:**

NSUN4‐mediated upregulation of SMURF1 promoted mitochondrial fission to accelerate DN progression via increasing CAMK1 ubiquitination. The discovery of the NSUN4/SMURF1/CAMK1 axis provides new insights into DN pathogenesis.

## Graphical abstract

NSUN4‐mediated upregulation of SMURF1 promoted mitochondrial fission and inhibited mitochondrial fusion to accelerate DN progression via increasing CAMK1 ubiquitination.

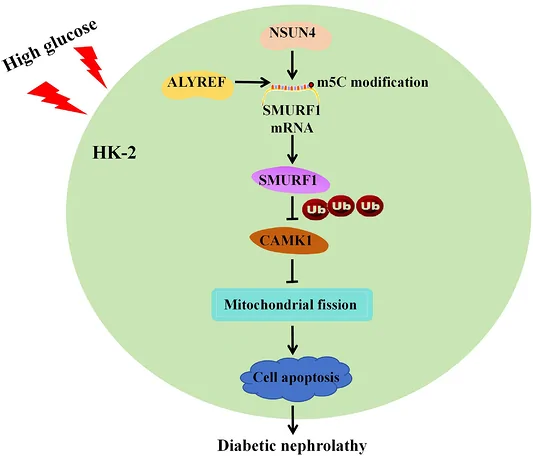

## 1 INTRODUCTION

The incidence of diabetes and its complications has been on the rise over the past 20 years.^1^ According to incomplete statistics, about 30%–40% of diabetes patients can develop diabetic nephropathy (DN).^2^, ^3^ Mitochondria, as energy factories in cells, play a key role in maintaining cell homeostasis and substance metabolism.^4^ Renal tubule injury is the early characteristic of DN, which may be due to mitochondrial dysfunction in proximal renal tubular epithelial cells.^5^ Importantly, it has been reported that mitochondrial fusion and fission imbalance are closely related to DN development.^6^ Therefore, it is necessary to reveal the potential mechanisms affecting mitochondrial dynamics to provide new ideas for DN treatment.

5‐methylcytosine (m5C) is a common post‐transcriptional modification of eukaryotic RNA involved in regulating many biological processes.^7^ m5C modification is reported to play an important role in human diseases, such as cardiovascular diseases,^8^ cancer,^9^ and central nervous system diseases.^10^ Besides, m5C modification is also related to diabetes complications progression.^11^ The regulators of m5C modification include m5C writers, m5C erasers and m5C readers.^12^ NOP2/Sun RNA methyltransferase family member 4 (NSUN4) belongs to NSUN family of m5C writers. Moreover, NSUN4 exists in mitochondria, and its expression disorder affects mitochondrial function.^13^ Wang et al. showed that NSUN4 was upregulated in diabetic retinopathy samples.^11^ However, whether NSUN4 is involved in DN progression and whether NSUN4‐mediated m5C modification regulates DN progression by controlling mitochondrial dynamics is unknown.

Epigenetic modifiers such as E3 ligases have been found to have a potential positive effect on DN treatment by regulating gene expression via linking ubiquitin to histones.^14^ SMAD ubiquitination regulatory factor 1 (SMURF1) is one of the E3 ubiquitin ligases.^15^ Lin et al.^16^ reported that knockout of SMURF1 could mitigate renal injury in streptozotocin (STZ)‐induced DN mouse models by inhibiting ubiquitination of TGR5. Besides, SMURF1 has been confirmed to be related to mitochondrial apoptotic.^17^ However, it is not clear whether NSUN4 regulates SMURF1 expression to affect mitochondrial dynamics and mediate DN progression. Besides, our previous studies confirmed the low calcium/calmodulin‐dependent protein kinase 1 (CAMK1) expression in DN patients, which suppressed mitochondrial fission to alleviate DN progression.^18^ Here, UbiBrowser 2.0 software predicted that SMURF1 was an ubiquitination ligase of CAMK1, and further analysis showed that there had interaction between SMURF1 and CAMK1. Nevertheless, whether NSUN4 mediates mitochondrial dynamics through the SMURF1/CAMK1 axis to affect DN progression remains unexplored.

Based on the above background, we speculated that NSUN4‐mediated m5C modification of SMURF1 regulated renal tubule mitochondrial dynamics and DN progression by mediating CAMK1 ubiquitination. These findings may provide new insights into DN pathogenesis and the development of clinical treatment options.

## 2 METHODS

### 2.1 Tissue samples

**What is already known?**

1. Downregulation of NSUN4 alleviates DN by inhibiting mitochondrial fission in renal tubular cells.
2. NSUN4 stabilizes SMURF1 via ALYREF‐mediated m5C modification, thereby promoting CAMK1 ubiquitination and degradation.

What does this study add?

1. Mitochondrial fission in renal tubular cells plays a key role in the development of DN.
2. NSUN4 regulates the SMURF1/CAMK1 axis to promote mitochondrial fission and thereby accelerate the DN process.

What is the clinical significance?

1. NSUN4/SMURF1/CAMK1 could be potential targets for DN treatment.

**Highlights**

1. NSUN4 knockout inhibits mitochondrial fission to alleviate STZ‐induced kidney injury.
2. Silencing of NSUN4 suppresses HG‐induced HK‐2 cell apoptosis by repressing mitochondrial fission.
3. NSUN4 promotes SMURF1 expression by binding to m5C reader ALYREF.
4. SMURF1 decreases CAMK1 expression via ubiquitination.

Kidney tissue samples from 10 DN patients (diagnosed by renal biopsy, urine albumin‐creatinine ratio ≥30 mg/g) and 10 patients with mild glomerular lesions were obtained from the Second Xiangya Hospital of Central South University. The data of DN patients and patients with mild glomerular lesions were shown in Table 1. Each participant signed written informed consent. This study was approved by the Ethics Committee of Second Xiangya Hospital of Central South University (approved number: Z0277).

**TABLE 1:** The information of DN patients and patients with mild glomerular lesions.

Patients	Age (years)	Sex	Scr (µmol/L)	Proteinuria (g/24 h)	eGFR (mL/min/1.73 m^2^)	UACR (mg/g)	Diabetes duration (years)	Diabetes background
**Control group (patients with mild glomerular lesions)**								
1	58	M	78.2	.26	96.74	21.6	–	–
2	47	M	81.6	.18	107.83	15.8	–	–
3	55	F	52.9	.52	117.22	27.6	–	–
4	51	M	65.4	.27	98.17	23.1	–	–
5	46	F	79.1	.34	105.27	17.2	–	–
6	38	F	53.8	.22	114.36	16.6	–	–
7	50	M	77.3	.41	96.03	22.1	–	–
8	62	F	65.8	.37	113.69	25.4	–	–
9	36	F	75.2	.15	118.41	19.3	–	–
10	44	M	58.6	.44	101.52	23.9	–	–
**DN group (DN patients)**								
1	39	F	112.6	3.26	52.13	41.4	3.7	T2D
2	51	F	116.8	4.68	59.28	39.2	4.2	T2D
3	54	M	126.9	3.07	71.21	52.6	1.6	T1D
4	66	M	137.2	5.25	41.39	62.1	1.3	T2D
5	42	F	98.9	4.19	36.66	37.5	5.1	T1D
6	33	M	107.2	3.76	41.72	44.7	2.5	T1D
7	45	M	127.6	2.78	63.58	39.8	2.8	T2D
8	42	F	108.1	4.26	33.56	33.7	1.9	T2D
9	56	M	113.4	5.51	40.15	53.2	2.4	T1D
10	47	F	107.5	3.93	68.85	40.3	3.1	T2D

### 2.2 DN mouse models

All animal experiments were approved by the Ethics Committee of Second Xiangya Hospital of Central South University (Approved number: 20230326) and were performed in compliance with the ARRIVE guidelines.^19^ Male C57BL/6 mice (6–8 weeks, 20–24 g) were purchased from SJA Laboratory Animal Co., Ltd. (Hunan, China) and randomly divided into 4 groups (*n* = 8 per group). As previously described,^18^ mice were intraperitoneally injected with 50 mg/kg STZ (18883‐66‐4, Sigma‐Aldrich, St. Louis, MO, USA) for 5 days. Among them, blood glucose levels>16.7 mmol/L for 3 consecutive days were the standard for successful establishment of animal models. The control group was injected with the same dose of citrate buffer. To explore the effect of NSUN4 and CAMK1 on renal injury, STZ‐induced DN mice were injected with lentivirus shRNA against NSUN4/CAMK1 (sh‐NSUN4/sh‐CAMK1) in the kidneys, and named as the STZ+sh‐NSUN4 group and STZ+sh‐NSUN4+sh‐CAMK1 group. Starting on day 0, body weight and blood glucose levels were measured every 2 weeks six times. Also, blood urea nitrogen (BUN), serum creatinine (Cre), urine N‐acetyl‐beta‐D glucosamine (U‐NAG), and albumin creatinine ratio (ACR) levels in living mice were detected using related kits (EIABUN, Invitrogen, Carlsbad, CA, USA; ab65340/ab204705/ab241018, Abcam, Cambridge, MA, USA). After the last weighing, all the mice were euthanized, and the kidney tissue was collected.

db/db mice spontaneously develop hyperglycaemia, insulin resistance, and progressive DN, closely mimicking the human disease course. To enhance the generalizability of the conclusions, male C57BL/6 db/db mice and db/m mice (6–8 weeks, 25–30 g, SJA Laboratory Animal Co., Ltd.) were used as a genetic model of type 2 diabetes.

### 2.3 Conditional knockout (cKO) of NSUN4 mouse model

Using the Cre‐LoxP recombination strategy, NSUN4 conditional knockout (NSUN4‐cKO) in proximal tubular cells was established by Gempharmatech (Jiangsu, China), and Cre‐negative littermate wild‐type (WT) mice served as the control (NSUN4‐WT) group. Briefly, NSUN4 heterozygous Flox mice were mated with Cre mice to generate NSUN4 Flox heterozygous and Cre‐positive mice. Then, they were mated with NSUN4 heterozygous Flox mice to generate NSUN4 Flox homozygous and Cre‐positive NSUN4‐cKO mice. In the present study, exon 4 of the NSUN4 gene was modified by LoxP to be excised by specific Cre mice. Following, mice were injected with STZ to construct the STZ+NSUN4‐WT group and STZ+NSUN4‐cKO group (*n* = 8/group). Mice's body weight, blood glucose, BUN, serum Cre, U‐NAG, and ACR levels in each group were assessed as described above.

### 2.4 Cell culture, treatment and transfection

Human HK‐2 cells (CL‐0109, Procell, Wuhan, China) were cultured in MEM containing 10% fetal bovine serum‌. Cells were treated with 30 mM D‐glucose for 48 h to mimic high glucose (HG) models,^18^, ^20^ and normal glucose (NG, 5.5 mM D‐glucose) treatment was used as a control. NSUN4/ALYREF/SMURF1/CAMK1 shRNAs (sh‐NSUN4: 5′‐CCGGCGGCAATAAGAAGTAGAAGATCTCGAGATCTTCTACTTCTTATTGCCGTTTTTG‐3′; sh‐ALYREF: 5′‐CCGGCGTGGAGACAGGTGGGAAACTCTCGAGAGTTTCCCACCTGTCTCCACGTTTTTG‐3′; sh‐SMURF1: 5′‐CCGGGCCCAGAGATACGAAAGAGATCTCGAGATCTCTTTCGTATCTCTGGGCTTTTTG‐3′; sh‐CAMK1: 5′‐CCGGCTACCACTCCTCACTGCATTTCTCGAGAAATGCAGTGAGGAGTGGTAGTTTTTG‐3′) were transfected using Lipofectamine 3000 (L3000150, Invitrogen) for 48 h before HG treatment. Moreover, pcDNA vectors were constructed by inserting the PCR products of NSUN4 (GenBank NM_199044.4)/ALYREF (GenBank NM_005782.4)/SMURF1 (GenBank NM_020429.3) into the pcDNA3.1(+) vector (V79020, Invitrogen), in which the CMV promoter was used to drive the expression of the target gene.

### 2.5 Detection of m5C level by LC‐MS/MS

Total RNAs were extracted by TRIzol reagent (15596018CN, Invitrogen). Purified RNA was digested using nuclease P1 and treated with NH_4_HCO_3_/alkaline phosphatase. After filtering, samples were analyzed by an HPLC‐QQQ‐MS/MS system as previously described.^21^

### 2.6 m5C dot blot assay

After extracted total RNAs, mRNA was purified using Dynabeads mRNA Purification Kit (61006, Invitrogen). Then, mRNA was denatured, labelled on Hybond‐N+ membranes, cross‐linked, sealed, and incubated with anti‐m5C (ab10805, Abcam) overnight. After that, the membrane was incubated with Goat anti‐mouse IgG (ab205719, Abcam) and visualized with an imaging system.

### 2.7 qRT‐PCR

After extracted by TRIzol reagent, total RNAs were reverse‐transcribed into cDNA with RevertAid RT Kit (K1691, Thermo Fisher Scientific, Waltham, MA, USA). Finally, qRT‐PCR was performed using SYBR Green (A25743, Thermo Fisher Scientific) with specific primers (Table 2). Relative mRNA expression was measured with the 2^−ΔΔCt^ method.

**TABLE 2:** Primer sequences used for qRT‐PCR.

Name	Forward (5′–3′)	Reverse (5′–3′)
Human NOP2	AAGCTAGGTCCGGATTGCAC	CCATGGTACAGAATGCGGGT
Human NSUN2	ACAGCGGTGAAGAGTTTGA	CTTTGCCAGGTCCTTTGCT
Human NSUN3	AGCTGAAAGCAAAATCAGAGGG	GAATCGGTTAAGCAGGACAGC
Human NSUN4	GCTGAGTGCCAAGGATTT	GGAGGGAAGCGACTGATA
Human NSUN5	CGCTACCATGAGGTCCACTAC	GCATCTCGCACCACGTCTT
Human NSUN6	GAAAGGCATGGGCATAAGAA	TGTGTGTTGTTTTCCCTCCA
Human NSUN7	TCATCTCCCAACTCACTTC	CAGGGGTTCATTCCCATAC
Human SMURF1	CTGGCAAGCGGTGGAGAC	CCGGTTAAAGCAGGTATGGG
Human CAMK1	CTCGGGTCATGATCCTCTGC	GCAGAGAAACTCCCGGTTCA
Human β‐actin	CTCCATCCTGGCCTCGCTGT	GCTGTCACCTTCACCGTTCC
Mice NOP2	AGCCTTCAGTTATCATGGGGC	TTTTGAATTGGTGCTGGGCG
Mice NSUN2	GCTGCTATCTGCTCGTCCAT	ACGTTTGTTCCACGGCATTG
Mice NSUN3	GACCCGGAAGTATGGCGTTT	AACACACTCAGCACGGTTCC
Mice NSUN4	TTGTTAGTTGACATGGCTGCG	TGGCTTCACTGACGAAGTCC
Mice NSUN5	CAAGGTTCATCGGGGTGTGA	CGTGGTACCTGGTAGGCTTG
Mice NSUN6	TCAGAGTGGATCTAGGCGCT	AGTTCTGGAATGAGCAGGGC
Mice NSUN7	CAAAGGGCCTCAACTTTGCC	AAGCCTTCACAGAGTGGACG
Mice SMURF1	AAGGCTCTGCAAGGCTCTAC	CTGCAAAGCCACAGGTTTCC
Mice CAMK1	AGGACTCAGAAACTGGTGGC	AGACGGCAATCTCGTTCTCC
Mice β‐actin	GGACTGTTACTGAGCTGCGTT	CGCCTTCACCGTTCCAGTT

### 2.8 Western blot (WB)

Proteins were extracted using RIPA buffer (P0013B, Beyotime, Shanghai, China), separated by SDS‐PAGE gel, and transferred to PVDF membranes. Membrane was incubated with anti‐NSUN4 (1:1000, ab101625, Abcam), anti‐mitofusin 1 (Mfn1) (1:1000, 13798‐1‐AP, Proteintech, Rosemont, IL, USA), anti‐mitofusin 2 (Mfn2) (1:2000, 12186‐1‐AP, Proteintech), anti‐fission 1 (Fis1) (1:1000, 10956‐1‐AP, Proteintech), anti‐phospho‐dynamin related protein 1 (p‐Drp1) Ser616 (1:1000, AF8470, Affinity, Jiangsu, China), anti‐dynamin related protein 1 (Drp1) (1:2000, 12957‐1‐AP, Proteintech), anti‐ALYREF (1:10000, ab202894, Abcam), anti‐SMURF1 (1:1000, ab300408, Abcam), anti‐CAMK1 (1:1000, 24312‐1‐AP, Proteintech), anti‐β‐actin (1:1000, ab8227, Abcam) or secondary antibody (1:10000, SA00001‐2, Proteintech). After that, protein signals were evaluated using ECL reagent (P0018S, Beyotime).

### 2.9 Immunofluorescence (IF) staining

HK‐2 cells under HG or NG conditions were fixed with 4% paraformaldehyde, permeated with 0.25% Triton X‐100, and blocked with goat serum. After incubation with anti‐NSUN4 (1:200, PA5‐140975, Invitrogen) and secondary antibodies, nuclei were stained with DAPI solution. The expression location of NSUN4 was analyzed under a fluorescence microscope.

### 2.10 Histological and immunohistochemical (IHC) staining

After being fixed with paraformaldehyde, mouse kidney tissue was embedded and cut into 4 µm sections. Sections were stained with a haematoxylin–eosin (H&E) staining kit (C0105S, Beyotime) and a periodic acid‐schiff (PAS) staining kit (C0142S, Beyotime), respectively. Finally, renal tubular damage score and glomerular damage score were carried out using a semiquantitative scoring system (0–3 points) to evaluate renal injury as previously described.^22^

For detecting NSUN4 expression, sections were stained with an SP kit (SP0041, Solarbio, Beijing, China) with anti‐NSUN4 (ab235430, Abcam). The positive cells of NSUN4 were observed under a microscope.

### 2.11 Apoptosis evaluation

Sections of mouse kidney tissues were dewaxed, digested with protease K, and sealed using 3% H_2_O_2_ in PBS. Besides, HK‐2 cells were fixed, permeated and sealed. After that, samples were incubated with TUNEL solution (C1086, Beyotime) and DAPI solution. A fluorescence microscope was used to measure TUNEL‐positive cells in five random fields.

### 2.12 Detection of ROS production

Mice frozen kidney tissue sections were treated with 10 µM dihydroethidium (DHE) working solution (HY‐D0079, MCE, Monmouth Junction, NJ, USA). After incubation, ROS production was evaluated by observing DHE fluorescence signal using a fluorescence microscope.

Frozen kidney sections from cells or mice were incubated with 5 µM MitoSOX Red solution (M36008, Invitrogen) for 30 min. Fluorescence was visualized using a fluorescence microscope to analyze mitochondrial ROS levels.

### 2.13 ATP detection

Kidney tissues or cells were lysed and centrifuged, and supernatants were collected. The supernatant was incubated with 100 µL of ATP assay working solution (S0026, Beyotime), and then the ATP level was measured by a luminometer.

### 2.14 Transmission electron microscope (TEM)

The obtained mouse kidney tissue was fixed, dehydrated, embedded, and sliced with an ultra‐micro microtome. Finally, sections were stained with uranyl acetate and lead citrate. Mitochondrial morphological changes (expressed as percentages, including mitochondrial length and mitochondrial fragmentation number) were observed by TEM in five random fields.

### 2.15 JC‐1 staining

Cells were incubated with JC‐1 solution (50‐113‐7449, Molecular Probes, Eugene, OR, USA). After incubation, red/green fluorescence was visualized using a fluorescence microscope to evaluate mitochondrial membrane potential (MMP) depolarization.

### 2.16 EdU assay

Cells were stained using EdU solution (E607204, Sangon, Shanghai, China), fixed with paraformaldehyde, and permeated with Triton X‐100. After that, the cell nucleus was stained with DAPI solution, and fluorescent images were captured under a microscope to calculate EdU‐positive cells (%).

### 2.17 RNA pull‐down assay

Biotin‐labelled RNA SMURF1 fragments with or without m5C modification (Oligo‐C or Oligo‐m5C) were synthesized by GeneCreate (Wuhan, China). Using the magnetic RNA‐protein pull‐down kit (20164, Thermo Fisher Scientific), cells were transfected with oligo‐C/oligo‐m5C, and cell supernatant was incubated with anti‐ALYREF (ab202894, Abcam) and magnetic beads. The resulting complexes were collected to measure ALYREF protein signal via WB. Untreated cell lysates were used for WB as input positive controls.

### 2.18 Methylated RNA immunoprecipitation (MeRIP)

According to the instructions of the m5C MeRIP Kit (GS‐ET‐001A, Cloud‐seq, Shanghai, China), the m5C modification level of SMURF1 was assessed. In short, total RNA was extracted and fragmented using fragmentation buffer. Fragmented RNA was incubated with Protein G magnetic beads precoated with anti‐m5C (ab10805, Abcam) or anti‐IgG (ab172730, Abcam). The m5C level of SMURF1 was assessed by qRT‐PCR.

### 2.19 RNA immunoprecipitation (RIP) assay

Using the Magna RIP kit (17‐700, Millipore, Billerica, MA, USA), HK‐2 cell lysates were incubated with anti‐ALYREF (ab202894, Abcam), anti‐IgG (ab172730, Abcam), and magnetic beads. After that, SMURF1 enrichment was detected by qRT‐PCR.

### 2.20 mRNA stability assay

The experimental principle of Actinomycin D (Act D) is to observe the degradation of the target mRNA by inhibiting the synthesis of RNA, so as to evaluate its stability. The WT or mutant‐type (MUT) plasmids of ALYREF m5C‐binding sequences (pc‐ALYREF‐WT or pc‐ALYREF‐MUT) were constructed by Genepharma (Shanghai, China). HG‐induced HK‐2 cells were transfected with sh‐NC/sh‐NSUN4/pc‐ALYREF‐WT/pc‐ALYREF‐MUT and then incubated with Act D (A9415, Sigma‐Aldrich). Afterwards, RNA was extracted at fixed time points, and qRT‐PCR was performed to assess SMURF1 mRNA stability.

### 2.21 Co‐IP and ubiquitination assay

UbiBrowser 2.0 software (http://ubibrowser.bio‐it.cn/ubibrowser_v3/) was used to predict the ubiquitination of CAMK1. HK‐2 cell lysates were incubated with anti‐SMURF1 (ab300408, Abcam), anti‐CAMK1 (24312‐1‐AP, Proteintech), anti‐IgG (ab172730, Abcam), and protein A/G‐agarose beads (78610, Invitrogen). The precipitates were eluted for measuring SMURF1 and CAMK1 protein levels by WB.

HK‐2 cells were transfected with HA‐Ub, pc‐SMURF1 or pc‐CAMK1, and cell lysates were incubated with anti‐HA (ab314237, Abcam), anti‐CAMK1 (24312‐1‐AP, Proteintech) and protein A/G agarose beads (78610, Invitrogen). Ub and CAMK1 protein signals were examined by WB.

### 2.22 Cycloheximide (CHX) assay

HK‐2 cells were transfected with sh‐NC/sh‐SMURF1 and treated with HG. Then, cells were incubated with CHX (25 µg/mL, C4859, Sigma‐Aldrich). At each time point, CAMK1 protein levels were tested using WB.

### 2.23 Data and statistical analysis

All cell experiments were repeated three times unless otherwise stated. All data are shown as mean ± SD using GraphPad Prism 8.0 software. Statistical significance was analyzed by Student's *t*‐test or ANOVA with Tukey's post hoc test. *p *< .05 was considered statistically significant.

## 3 RESULTS

### 3.1 The high expression of NSUN4 increased m5C level in DN

**FIGURE 1:**
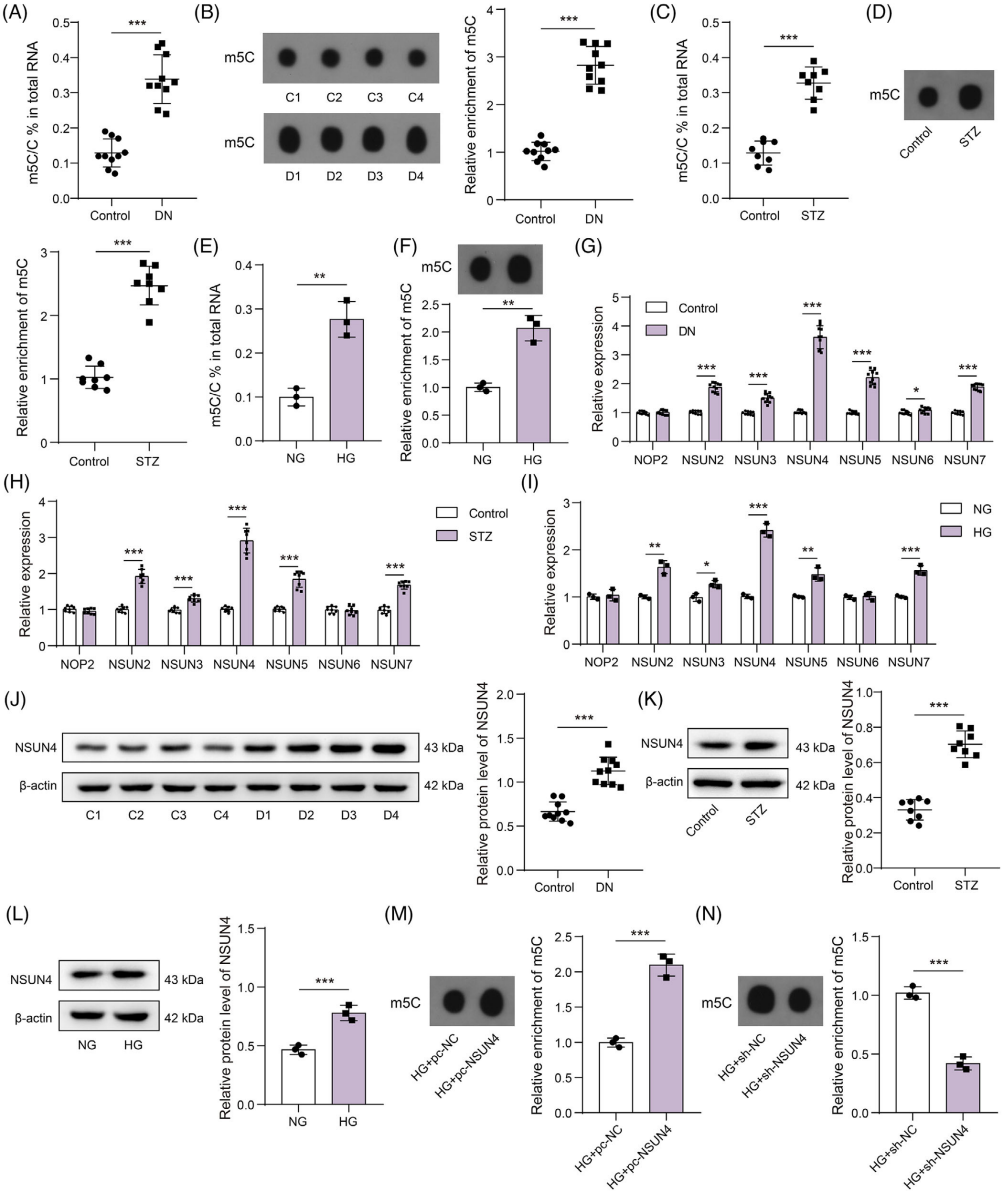
Effect of NSUN4 on m5C level in DN. (A–F) m5C level was detected with LC‐MS/MS and dot blot assay in DN patients (*n* = 10), STZ‐induced DN mouse models (*n* = 8), and HG‐induced HK‐2 cells (*n* = 3). (G–I) The expression levels of 7 m5C writers were tested by qRT‐PCR in DN patients (*n* = 10), mouse models (*n* = 8), and cell models (n = 3). (J–L) WB was used to measure NSUN4 protein expression in DN patients (*n* = 10), mouse models (*n* = 8), and cell models (*n* = 3). (M, N) m5C expression was checked using dot blot assay in HG‐induced HK‐2 cells transfected with pc‐NC/pc‐NSUN4/sh‐NC/sh‐NSUN4 (*n* = 3). The data are presented as the mean ± SD. (A–N) Student's *t*‐test. *p < .05, **p < .01, ***p < .001.

Given the important role of m5C modification in DN,^11^ we measured m5C level in kidney tissues and cells. By LC‐MS/MS and dot blot analysis, m5C level was elevated in the kidney tissues of DN patients and STZ‐induced DN mouse models (Figure 1A–D). Moreover, we also detected a high m5C level in HG‐induced HK‐2 cells (Figure 1E,F). To confirm the m5C writers that regulate m5C levels in DN, we analyzed the expression of 7 m5C writers using qRT‐PCR and discovered that NSUN2/3/4/5/7 were upregulated in DN patients, mouse models, and cell models, among which NSUN4 was the most significant (Figure 1G–I). Therefore, we selected NSUN4 for the follow‐up study. At the protein level, NSUN4 expression was also markedly enhanced in DN patients, mouse models, and cell models (Figure 1J–L). Similarly, compared with db/m mice, the NSUN4 mRNA and protein expression was significantly upregulated in db/db mice (Figure S1A,B). Also, NSUN4 was expressed in the cytoplasm, and its expression was increased in HK‐2 cells after HG induction (Figure S2). NSUN4 overexpression increased m5C levels, and NSUN4 knockdown decreased m5C levels in HG‐induced HK‐2 cells (Figure 1M,N). These data showed that NSUN4 positively regulated m5C levels in DN.

### 3.2 NSUN4‐cKO alleviated renal injury in DN mice

**FIGURE 2:**
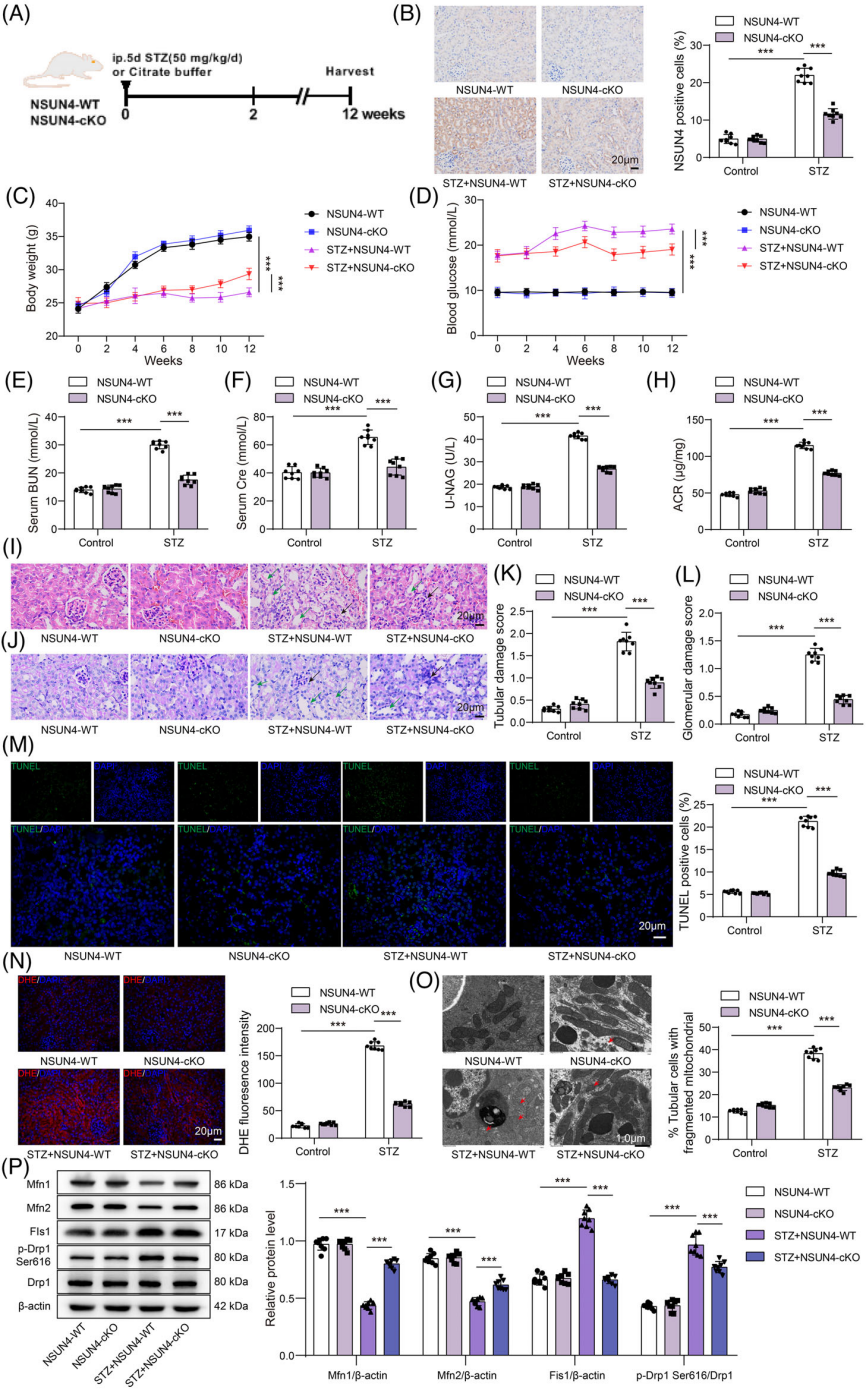
Effect of NSUN4‐cKO on renal injury and mitochondrial fission in STZ‐induced DN mice. (A) The flow chart of the model construction. (B) NSUN4 expression was measured using IHC staining in the kidney tissues of NSUN4‐WT, NSUN4‐cKO, STZ+NSUN4‐WT and STZ+NSUN4‐cKO groups. (C, D) Statistical chart of body weight and blood glucose changes for 12 consecutive weeks. (E–H) Serum BUN, serum Cre, U‐NAG, and ACR levels were detected with related kits. (I–L) Renal tubular damage score and glomerular damage score were assessed to evaluate renal injury by H&E and PAS staining. (M) Cell apoptosis was tested by TUNEL staining. (N) DHE staining was used to assess ROS production. (O) Representative image of fragmented mitochondria observed by TEM. (P) Mfn1, Mfn2, Fis1, p‐Drp1 Ser616, and Drp1 protein levels were evaluated using WB. The data are presented as the mean ± SD. (B, E–H, K–P) One‐way ANOVA with Tukey's post hoc test; (C, D) two‐way ANOVA with Tukey's post hoc test. *n* = 8, *p < .05, **p < .01, ***p < .001.

Following this, we constructed an NSUN4‐WT/cKO mouse model to evaluate the effect of NSUN4 knockout on renal injury in DN. The flow chart of the model construction is shown in Figure 2A. IHC assay showed that NSUN4 in the proximal tubule of STZ‐induced DN mice was significantly increased, and NSUN4‐cKO decreased NSUN4 expression (Figure 2B). STZ‐induced DN mice had lower body weight and higher blood glucose than non‐diabetic NSUN4‐WT/cKO mice (Figure 2C,D). Compared with the non‐diabetic NSUN4‐WT/cKO group, serum BUN, serum Cre, U‐NAG, and ACR levels in STZ‐induced DN mice were increased, while NSUN4‐cKO reduced these levels in DN mice (Figure 2E–H). Besides, H&E and PAS staining further confirmed that NSUN4‐cKO reduced renal tubular damage score and glomerular damage score of STZ‐induced DN mice (Figure 2I–L). Also, TUNEL‐positive cells, ROS production, and mitochondrial ROS levels were enhanced, and ATP levels were reduced in the kidney tissue of STZ‐induced DN mice, while these effects were reversed in the diabetic NSUN4‐cKO group (Figure 2M,N; Figure S3A,B). Meanwhile, TEM observed that NSUN4‐cKO decreased the ratio of fragmented mitochondria in STZ‐induced DN mice, as indicated by increased mitochondrial length, reduced mitochondrial fragmentation, and enhanced the aspect ratio (Figure 2O; Figure S3C). Besides, NSUN4‐cKO increased the protein levels of mitochondrial fusion markers (Mfn1 and Mfn2) and decreased mitochondrial fission markers (Fis1 and p‐Drp1 Ser616/Drp1) in STZ‐induced DN mice (Figure 2P). All data suggested that NSUN4 knockout repressed mitochondrial fission and promoted mitochondrial fusion to alleviate renal injury in DN mouse models.

### 3.3 Knockdown of NSUN4 inhibited HG‐induced HK‐2 cell mitochondrial fission and apoptosis

**FIGURE 3:**
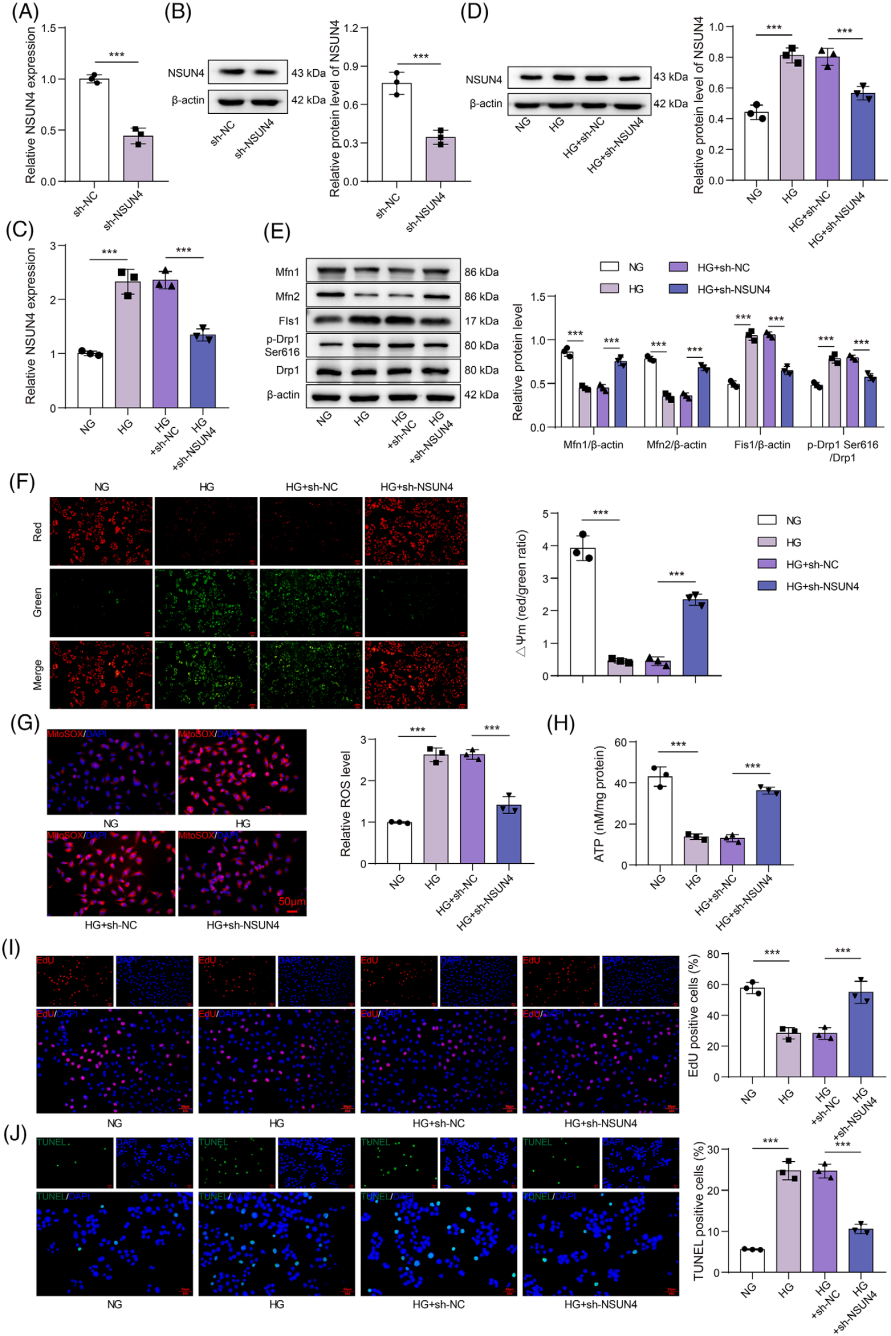
Effect of sh‐NSUN4 on HG‐induced HK‐2 cell apoptosis and mitochondrial fission. (A, B) NSUN4 mRNA and protein expression were tested with qRT‐PCR and WB in HK‐2 cells transfected with sh‐NC/sh‐NSUN4. (C–J) HG‐induced HK‐2 cells were transfected with sh‐NC/sh‐NSUN4. (C, D) qRT‐PCR and WB were performed to measure NSUN4 mRNA and protein expression. (E) Mfn1, Mfn2, Fis1, p‐Drp1 Ser616, and Drp1 protein expression was detected using WB. (F) MMP depolarization was checked with JC‐1 staining. (G) Mitochondrial ROS level was examined by MotiSOX Red. (H) ATP level was tested using an ATP assay kit. (I, J) Cell proliferation and apoptosis were examined by EdU assay and TUNEL staining. The data are presented as the mean ± SD. (A, B) Student's *t*‐test; (C–J) one‐way ANOVA with Tukey's post hoc test. *n* = 3, *p < .05, **p < .01, ***p < .001.

Next, the effect of NSUN4 knockdown on HG‐induced HK‐2 cell mitochondrial fission and apoptosis was explored. NSUN4 mRNA and protein expression were reduced by approximately 60% after transfection with sh‐NSUN4 (Figure 3A,B). Also, the transfection of sh‐NSUN4 decreased NSUN4 mRNA and protein levels in HG‐induced HK‐2 cells (Figure 3C,D). Silencing of NSUN4 promoted Mfn1 and Mfn2 levels, while inhibited Fis1 and p‐Drp1 Ser616/Drp1 levels (Figure 3E). Besides, sh‐NSUN4 increased the red/green fluorescence intensity ratio of JC‐1 (Figure 3F), indicating that NSUN4 promoted MMP depolarization. Also, sh‐NSUN4 decreased mitochondrial ROS levels and increased ATP levels (Figure 3G,H). Meanwhile, sh‐NSUN4 facilitated EdU‐positive cells and repressed TUNEL‐positive cells (Figure 3I,J). All results indicated that NSUN4 might promote mitochondrial fission to accelerate cell apoptosis.

### 3.4 NSUN4/ALYREF promoted SMURF1 m5C modification

**FIGURE 4:**
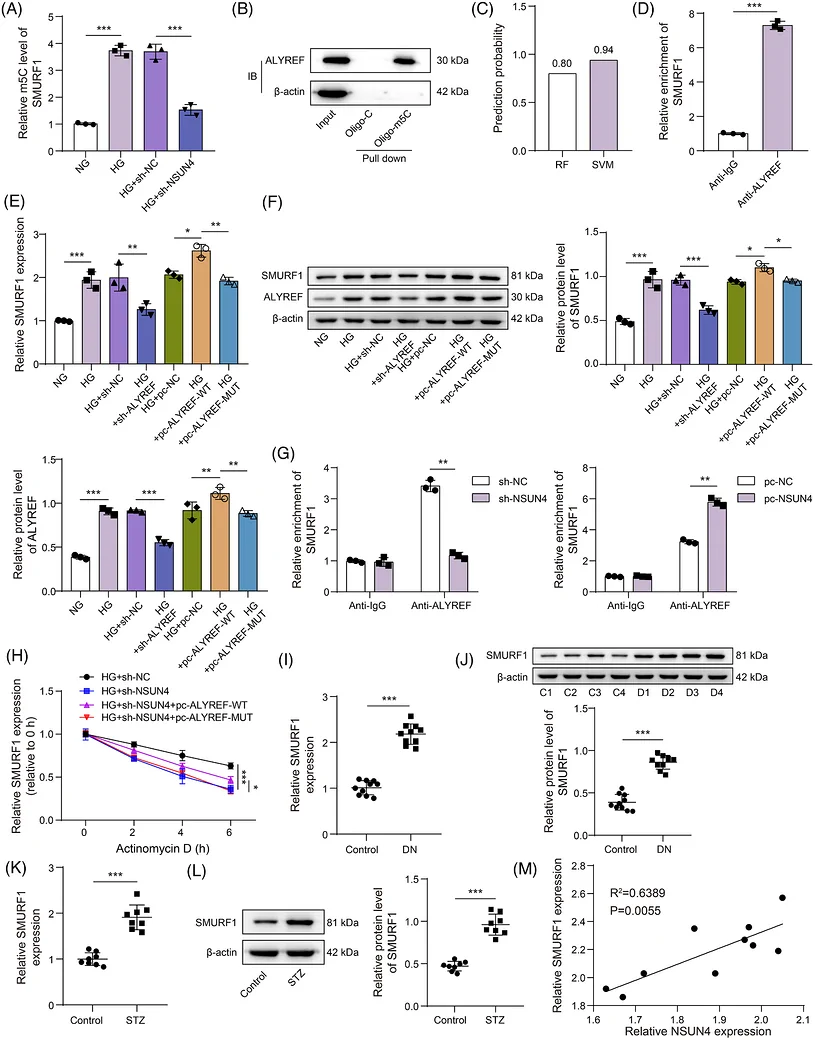
Effects of NSUN4 and ALYREF on SMURF1 mRNA stability. (A) MeRIP assay was used to assess the m5C modification level of SMURF1 in HG‐induced HK‐2 cells transfected with sh‐NC/sh‐NSUN4. (B) The relationship between ALYREF and m5C modification was evaluated using an RNA pull‐down assay (*n* = 3). (C, D) The interaction of ALYREF and SMURF1 was measured by RPISeq (http://pridb.gdcb.iastate.edu/RPISeq/) (both RF and SVM scores greater than .5 represent a possible interaction) and RIP assay (*n* = 3). (E) SMURF1 mRNA expression was examined with qRT‐PCR in each group (*n* = 3). (F) SMURF1 and ALYREF protein levels were evaluated using WB in each group (n = 3). (G) RIP assay was used to assess the effect of NSUN4 on the interaction between ALYREF and SMURF1 (*n* = 3). (H) SMURF1 mRNA stability was tested using the Act D assay in each group (*n* = 3). (I–L) qRT‐PCR and WB were performed to detect SMURF1 mRNA and protein expression in kidney tissues of DN patients (*n* = 10) and STZ‐induced DN mouse models (*n* = 8). (M) The correlation between NSUN4 and SMURF1 was evaluated using the Pearson coefficient analysis in DN patients (*n* = 10). The data are presented as the mean ± SD. (A, E–G) One‐way ANOVA with Tukey's post hoc test; (D, I–L) Student's *t*‐test; (H) two‐way ANOVA with Tukey's post hoc test. *p < .05, **p < .01, ***p < .001.

It has been reported that m5C writer NSUN4 can interact with m5C reader ALYREF to regulate the expression of downstream factors.^21^ Given the vital function of SMURF1 in DN progression,^16^ we further explored whether NSUN4/ALYREF regulates SMURF1 through m5C modification to affect DN progression. HG treatment increased the m5C modification level of SMURF1, whereas sh‐NSUN4 reversed this effect (Figure 4A). Besides, the RNA pull‐down assay confirmed that the binding ability of ALYREF and oligo‐m5C was higher than that of the unmethylated control group (Figure 4B). RPISeq predicted that the Random Forest (RF) and Support Vector Machine (SVM) scores of ALYREF and SMURF1 were .80 and .94, respectively, indicating a high binding capacity between them (Figure 4C). Besides, SMURF1 enrichment was elevated by anti‐ALYREF, further verifying their interaction (Figure 4D). HG treatment enhanced SMURF1 mRNA and protein expression, and sh‐ALYREF reversed this effect. Meanwhile, the pc‐ALYREF‐WT vector could improve SMURF1 mRNA and protein expression (Figure 4E,F). Meanwhile, sh‐NSUN4 reduced the binding of ALYREF to SMURF1 mRNA, while pc‐NSUN4 had the opposite effect (Figure 4G). Furthermore, pc‐ALYREF‐WT partially eliminated the decreasing effect of sh‐NSUN4 on SMURF1 mRNA stability (Figure 4H). Also, SMURF1 mRNA and protein expression were upregulated in DN patients, STZ‐induced DN mouse models, and db/db mice (Figure 4I–L; Figure S1C,D). Moreover, NSUN4 expression was positively correlated with SMURF1 expression in DN patients (95% confidence interval: .445 to 1.853) (Figure 4M). Above all, NSUN4 increased SMURF1 mRNA stability by ALYREF‐mediated m5C modification.

### 3.5 SMURF1 overexpression reversed sh‐NSUN4‐mediated cell mitochondrial fission and apoptosis

**FIGURE 5:**
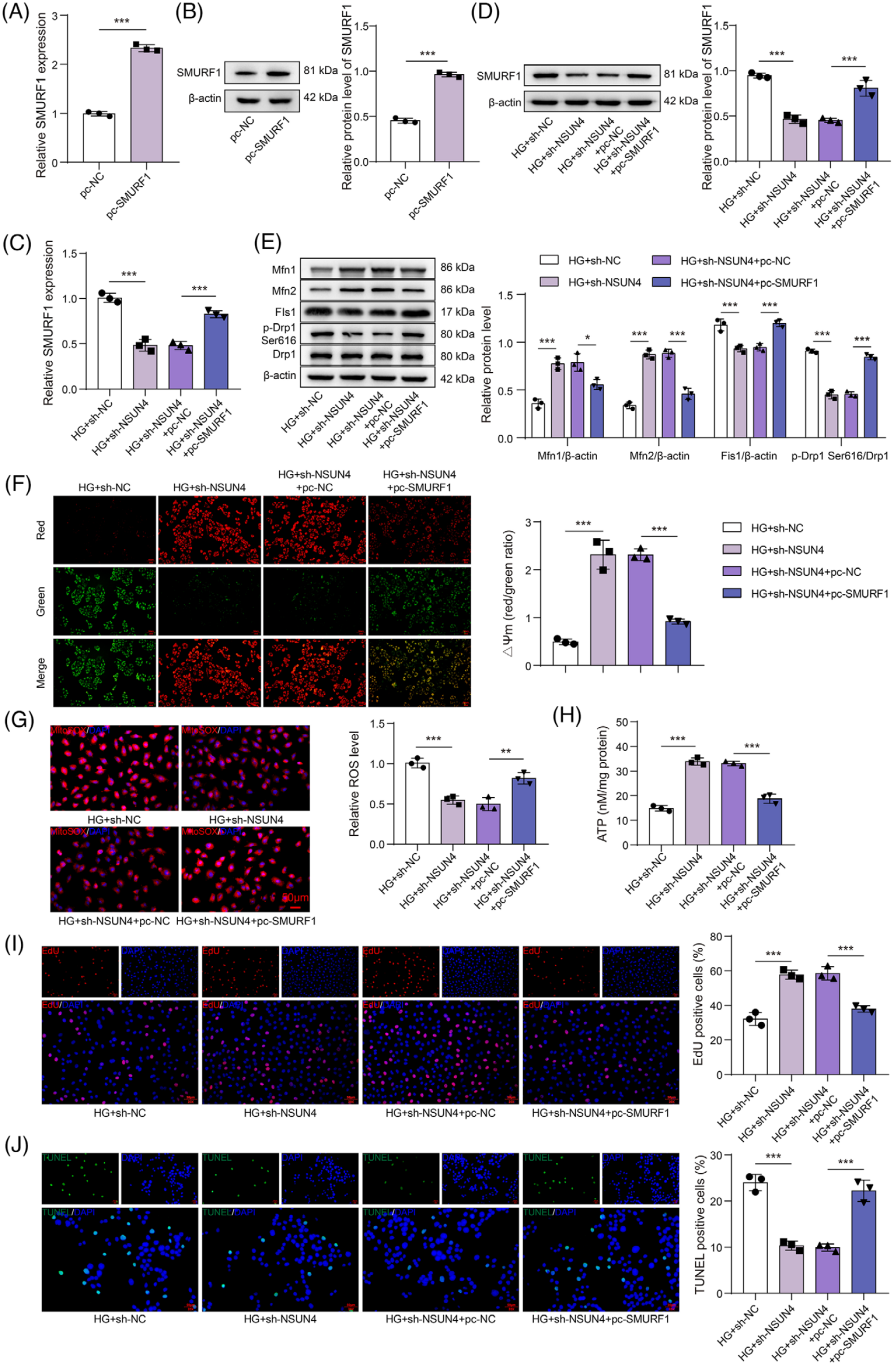
Effects of sh‐NSUN4 and pc‐SMURF1 on HG‐induced HK‐2 cell apoptosis and mitochondrial fission. (A‐B) SMURF1 mRNA and protein expression were detected using qRT‐PCR and WB in HK‐2 cells transfected with pc‐NC/pc‐SMURF1. (C–J) HG‐induced HK‐2 cells were transfected with sh‐NC/sh‐NSUN4/pc‐NC/pc‐SMURF1. (C–D) qRT‐PCR and WB were used to assess SMURF1 mRNA and protein expression. (E) WB was performed to measure Mfn1, Mfn2, Fis1, p‐Drp1 Ser616 and Drp1 protein expression. (F) MMP depolarization was examined by JC‐1 staining. (G) Mitochondrial ROS level was assessed by MotiSOX Red. (H) ATP level was measured with ATP Assay Kit. (I, J) Cell proliferation and apoptosis were checked using EdU assay and TUNEL staining. The data are presented as the mean ± SD. (A, B) Student's *t*‐test; (C–J) one‐way ANOVA with Tukey's post hoc test. *n* = 3, *p < .05, **p < .01, ***p < .001.

To investigate whether NSUN4 regulated cell mitochondrial fission and apoptosis by mediating SMURF1 expression, we performed rescue experiments. As shown in Figure 5A,B, the transfection of pc‐SMURF1 significantly increased SMURF1 mRNA and protein expression by approximately 100%‐130%, indicating successful transfection. After co‐transfection, pc‐SMURF1 reversed the decreasing effect of sh‐NSUN4 on SMURF1 expression (Figure 5C,D). Besides, pc‐SMURF1 abolished sh‐NSUN4‐mediated the increasing on Mfn1 and Mfn2 levels, as well as the decreasing on Fis1 and p‐Drp1 Ser616/Drp1 levels (Figure 5E). Also, the facilitation effect of sh‐NSUN4 on JC‐1 red/green fluorescence intensity ratio, EdU‐positive cells, and ATP levels, as well as the inhibition effect on TUNEL‐positive cells and mitochondrial ROS production, could be reversed by pc‐SMURF1 (Figure 5F–J). Therefore, NSUN4 might increase SMURF1 expression to enhance renal cell mitochondrial fission and apoptosis.

### 3.6 SMURF1 promoted the ubiquitination and degradation of CAMK1

**FIGURE 6:**
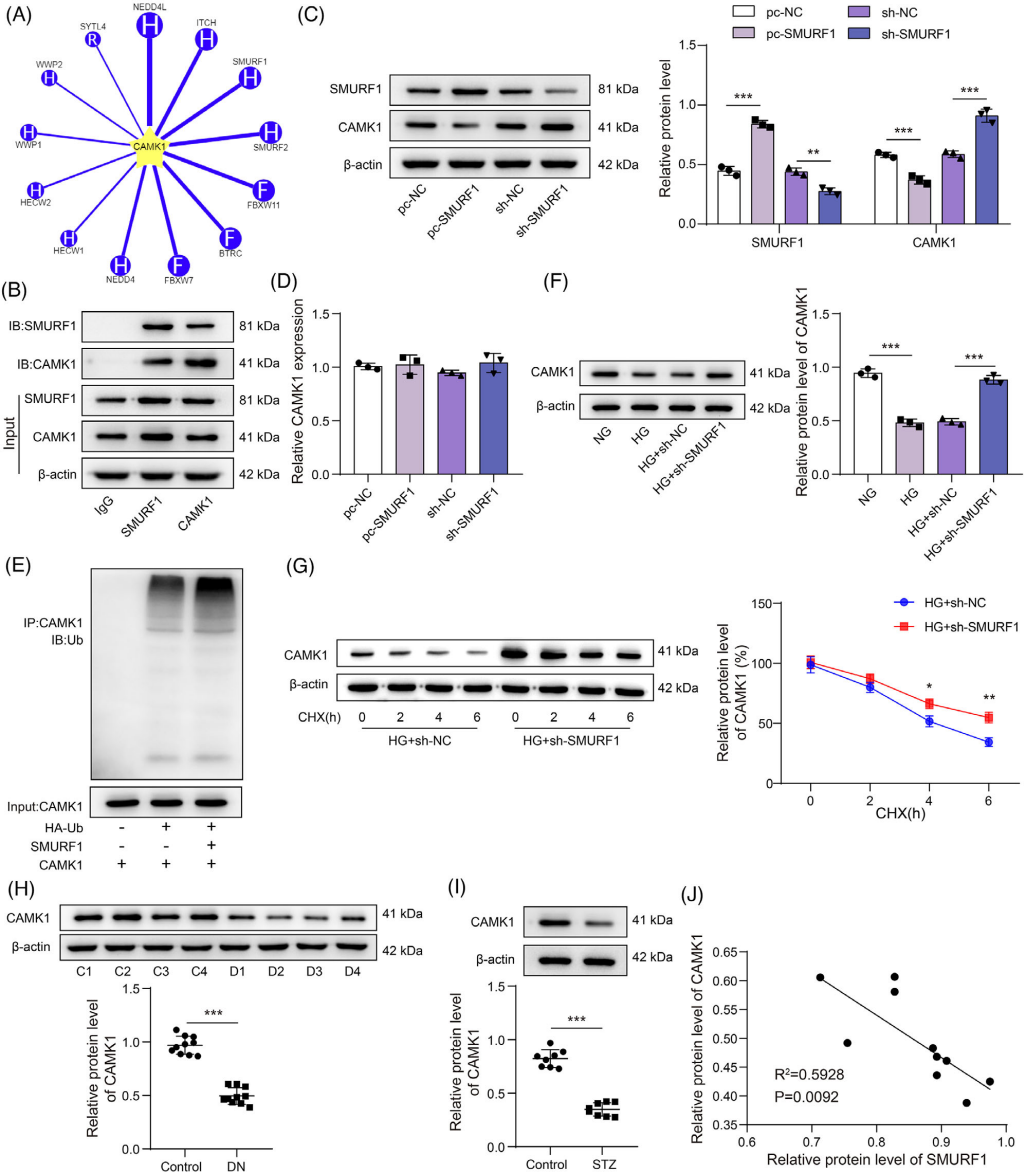
Effect of SMURF1 on CAMK1 ubiquitination. (A) UbiBrowser 2.0 (http://ubibrowser.bio‐it.cn/ubibrowser_v3/) prediction results diagram. (B) The relationship between SMURF1 and CAMK1 was evaluated using a Co‐IP assay (*n* = 3). (C) SMURF1 and CAMK1 protein expression was measured with WB in HK‐2 cells transfected with pc‐NC/pc‐SMURF1/sh‐NC/sh‐SMURF1 (*n* = 3). (D) CAMK1 mRNA expression was evaluated using qRT‐PCR (*n* = 3). (E) Ubiquitination assay was used to evaluate the effect of SMURF1 on the ubiquitination level of CAMK1 (*n* = 3). (F) CAMK1 protein expression was tested using WB in HG‐induced HK‐2 cells transfected with sh‐NC/sh‐SMURF1 (*n* = 3). (G) CHX assay was used to explore the effect of sh‐SMURF1 on CAMK1 protein stability. (H, I) CAMK1 protein expression was examined using WB in kidney tissues of DN patients (*n* = 10) and STZ‐induced DN mouse models (*n* = 8). (J) Pearson coefficient analysis was used to assess the correlation between SMURF1 and CAMK1 in DN patients (*n* = 10). The data are presented as the mean ± SD. (C–D, F) One‐way ANOVA with Tukey's post hoc test; (G) two‐way ANOVA with Tukey's post hoc test; (H, I) Student's *t*‐test. *p < .05, **p < .01, ***p < .001.

Our previous studies confirm that CAMK1 is a key molecule regulating mitochondrial dynamics affecting DN progression.^18^ Through UbiBrowser 2.0 software prediction, we found that there was an interaction between E3 ubiquitin ligase SMURF1 and CAMK1 (Figure 6A), and the Co‐IP assay further confirmed their interaction (Figure 6B). Besides, pc‐SMURF1 and sh‐SMURF1 could enhance and reduce SMURF1 protein levels by about 0.9‐fold and 0.4‐fold, while decreasing and increasing CAMK1 protein levels by about 0.4‐fold and 0.5‐fold, respectively, but had no effect on the CAMK1 mRNA expression (Figure 6C,D). Ubiquitination assay showed that SMURF1 overexpression facilitated the ubiquitination and degradation of endogenous CAMK1 (Figure 6E). Moreover, HG treatment markedly reduced CAMK1 protein levels, and sh‐SMURF1 further increased CAMK1 expression (Figure 6F). CHX assay showed that SMURF1 knockdown increased CAMK1 protein stability under HG conditions (Figure 6G). In addition, CAMK1 protein expression was downregulated in DN patients, STZ‐induced DN mouse models, and db/db mice (Figure 6H,I; Figure S1E). Importantly, SMURF1 expression was negatively correlated with CAMK1 expression in DN patients (95% confidence interval: −1.232 to −.239) (Figure 6J). All data revealed that SMURF1 decreased CAMK1 expression through ubiquitination.

### 3.7 SMURF1 knockdown inhibited mitochondrial fission and cell apoptosis by increasing CAMK1 expression

**FIGURE 7:**
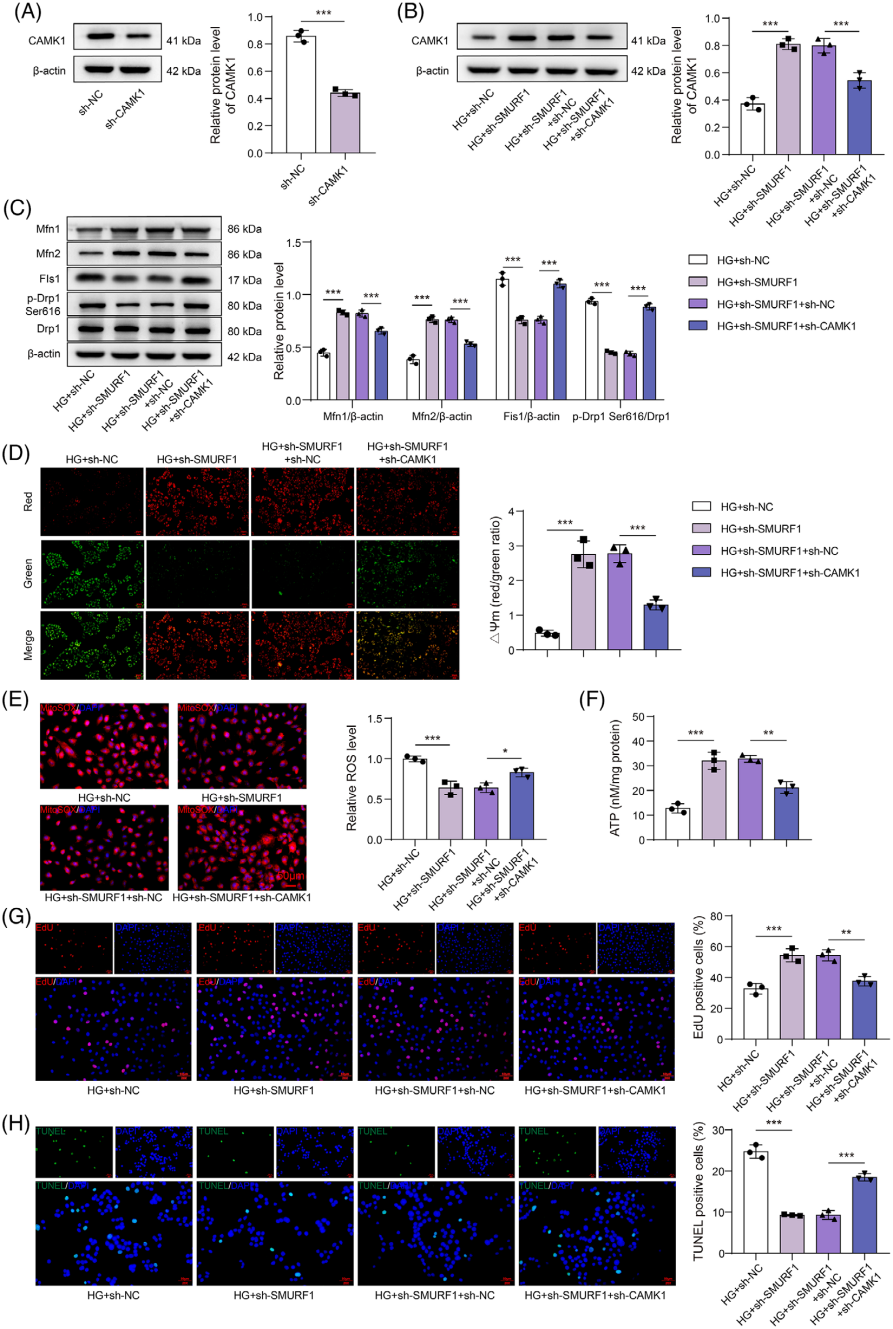
Effects of sh‐SMURF1 and sh‐CAMK1 on HG‐induced HK‐2 cell apoptosis and mitochondrial fission. (A) CAMK1 protein expression was tested with WB in HK‐2 cells transfected with sh‐NC/sh‐CAMK1. (B–H) HG‐induced HK‐2 cells were transfected with sh‐NC/sh‐SMURF1/sh‐CAMK1. (B) WB were used to test CAMK1 protein expression. (C) Mfn1, Mfn2, Fis1, p‐Drp1 Ser616, and Drp1 protein expression was detected using WB. (D) MMP depolarization was measured with JC‐1 staining. (E) Mitochondrial ROS level was examined by MotiSOX Red. (F) ATP level was assessed with an ATP assay kit. (G, H) EdU assay and TUNEL staining were performed to detect cell proliferation and apoptosis. The data are presented as the mean ± SD. (A) Student's *t*‐test; (B–H) one‐way ANOVA with Tukey's post hoc test. *n* = 3, *p < .05, **p < .01, ***p < .001.

To further explore whether SMURF1 regulated DN progression by mediating CAMK1 expression, rescue experiments were carried out. CAMK1 protein expression was reduced by approximately 50% after being transfected with sh‐CAMK1 (Figure 7A). In the co‐transfection system, sh‐CAMK1 reverted the sh‐SMURF1‐mediated increase in CAMK1 protein expression (Figure 7B). SMURF1 knockdown enhanced Mfn1 and Mfn2 levels, while reducing Fis1 and p‐Drp1 Ser616/Drp1 protein levels. However, these effects were reversed by sh‐CAMK1 (Figure 7C). Besides, sh‐SMURF1 promoted JC‐1 red/green fluorescence intensity ratio, EdU‐positive cells and ATP levels, while repressed TUNEL‐positive cells and mitochondrial ROS production, but sh‐CAMK1 also eliminated the above effects (Figure 7D–H). All data indicated that SMURF1 accelerated renal cell mitochondrial fission and apoptosis via downregulating CAMK1.

### 3.8 NSUN4 promoted renal injury in STZ‐induced DN mice by regulating SMURF1/CAMK1 axis

**FIGURE 8:**
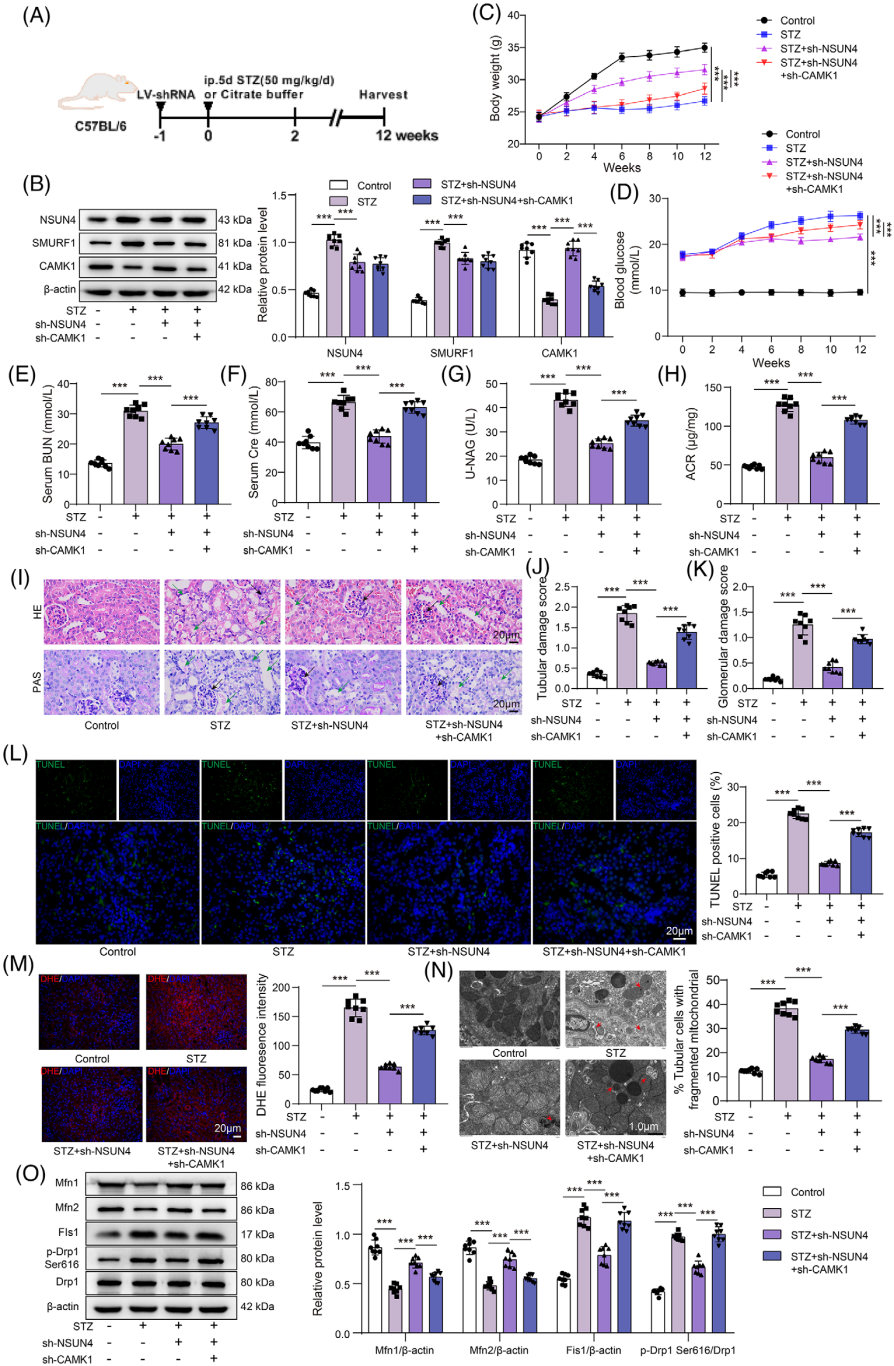
Effects of sh‐NSUN4 and sh‐CAMK1 on renal injury in STZ‐induced DN mice. (A) The flow chart of the model construction. (B) NSUN4, SMURF1, and CAMK1 protein expression was detected with WB in control, STZ, STZ+sh‐NSUN4, and STZ+sh‐NSUN4+sh‐CAMK1 groups. (C, D) Statistical chart of body weight and blood glucose changes for 12 consecutive weeks. (E–H) Related kits were used to test Serum BUN, serum Cre, U‐NAG, and ACR levels. (I–K) H&E and PAS staining were performed to evaluate renal tubular damage score and glomerular damage score for assessing renal injury. (L) Cell apoptosis was measured using TUNEL staining. (M) DHE staining was performed to check ROS production. (N) TEM was used to observe the representative image of fragmented mitochondria. (O) Mfn1, Mfn2, Fis1, p‐Drp1 Ser616, and Drp1 protein expression was assessed with WB. The data are presented as the mean ± SD. (B, E–H, J–O) One‐way ANOVA with Tukey's post hoc test; (C, D) two‐way ANOVA with Tukey's post hoc test. *n* = 8, *p < .05, **p < .01, ***p < .001.

To confirm whether NSUN4 regulated DN progression through the SMURF1/CAMK1 axis, we performed animal experiments. The flow chart of LV‐shRNA injection and model construction is shown in Figure 8A. The injection of sh‐NSUN4 decreased NSUN4 and SMURF1 and increased CAMK1 levels in STZ‐induced DN mice, while sh‐CAMK1 only further reduced CAMK1 expression (Figure 8B). Besides, sh‐NSUN4 mediated the increasing effect on body weight and the decreasing effect on blood glucose could be abolished by sh‐CAMK1 in DN mice (Figure 8C,D). Furthermore, sh‐CAMK1 reversed the inhibitory effect of sh‐NSUN4 on serum BUN level, serum Cre level, U‐NAG level, ACR level, renal tubular damage score, glomerular damage score, TUNEL‐positive cells, ROS production and mitochondrial ROS levels, as well as the enhancing effect on ATP levels (Figure 8E–M; Figure S4A,B). Also, sh‐NSUN4 increased the aspect ratio and reduced mitochondrial fragmentation, while sh‐CAMK1 abolished these effects (Figure 8N; Figure S4C). Meanwhile, sh‐CAMK1 reverted sh‐NSUN4‐mediated the decreasing on Fis1, p‐Drp1 Ser616/Drp1 levels, and the increasing on Mfn1 and Mfn2 levels (Figure 8O). These results indicated that NSUN4 enhanced DN mice renal injury by accelerating mitochondrial fission via regulating the SMURF1/CAMK1 axis.

## 4 DISCUSSION

Diabetes directly damages renal tubules and causes mitochondrial dysfunction, such as decreased biological energy, excessive production of mtROS, and autophagy defects.^23^ It had been reported that HIF‐1α protected tubular injury of DN by regulating mitochondrial dynamics mediated by HO‐1.^24^ Empagliflozin alleviated mitochondrial fission to inhibit diabetic renal tubular injury.^25^ These studies all suggest that mitochondrial dynamics is involved in the DN process. Our study explored a novel molecular mechanism mediating DN progression by affecting mitochondrial dynamics. Our results revealed that NSUN4 regulated the SMURF1/CAMK1 axis to accelerate DN progression by promoting mitochondrial fission.

m5C is a rich RNA modification in regulating gene expression,^26^ and is expected to become a new epigenetic mark.^27^, ^28^ For example, m5C score could be used as a reliable prognosis indicator for acute myeloid leukemia,^29^ and m5C modification might be a new strategy to improve treatment outcomes for cancer patients.^30^ In eukaryotes, m5C modification can be catalyzed by the NSUN family.^31^ The m5C writer NSUN4 is a recently discovered novel mitochondrial methyltransferase.^32^ Studies have found that NSUN4 promoted malignant progression of glioma by catalyzing CDC42 m5C modification.^21^ NSUN4‐mediated m5C modification of circERI3 changed mitochondrial energy metabolism, thus promoting the development of lung cancer.^33^ Although Wang et al.^11^ revealed high NSUN4 expression in diabetes‐related diseases, its role in DN progression remains unclear. Our study detected high m5C levels in DN patients, mouse models, and cell models, and identified NSUN4 as an upregulated m5C writer that significantly promoted m5C levels. The animal study revealed that NSUN4‐cKO could alleviate STZ‐induced renal injury by inhibiting renal tubular cell apoptosis and mitochondrial fission. Moreover, NSUN4 knockdown reduced HG‐induced mitochondrial fission, MMP depolarization, and apoptosis. The above data further suggested that NSUN4 might accelerate mitochondrial fission to facilitate DN mice renal injury and renal cell apoptosis, providing new evidence that NSUN4 acted as a potential therapeutic target for DN.

The regulation of downstream genes by NSUN4‐mediated m5C modification is achieved through interacting with m5C reader ALYREF.^21^ SMURF1 is an important homologous member of E6‐AP C‐terminal E3 ubiquitin ligase. It was reported that SMURF1 was higher in HG‐induced glomerular mesangial cells, and its knockdown repressed ROS production to improve renal function of diabetic mice.^34^ Moreover, SMURF1 knockout attenuated renal injury in diabetic mice.^16^ These data confirmed the positive role of SMURF1 in DN. This research found that NSUN4‐catalyzed m5C modification regulated the binding of ALYREF and SMURF1 mRNA, and NSUN4 promoted the mRNA stability of SMURF1. Through rescue experiments, SMURF1 overexpression eliminated sh‐NSUN4‐mediated inhibition of HG‐induced apoptosis and mitochondrial fission, confirming that NSUN4 increased SMURF1 expression to accelerate DN progression.

SMURF1 has been confirmed to regulate the ubiquitination and degradation of proteins.^35^ For example, SMURF1 interacted with kindin‐2 to promote its ubiquitination and degradation, thereby inhibiting integrin activation.^36^ Also, SMURF1 ubiquitinated and degraded PTEN to promote glioblastoma cell growth and colony formation.^37^ In DN, SMURF1 increased the ubiquitination and proteasomal degradation of TGR5 to accelerate renal injury.^16^ CAMK1 is activated by the intracellular Ca^2+^ pathway and is involved in many cellular functions.^38^ CAMK1 might regulate STZ‐induced renal injury in diabetic rats.^39^ Importantly, our previous study revealed that high CAMK1 levels suppressed mitochondrial fission to relieve STZ‐induced renal injury in diabetic mice.^18^ Consistent with our previous research,^18^ this study further confirmed the downregulated CAMK1 expression. Moreover, our results found that SMURF1 interacted with CAMK1 to inhibit its level by increasing its ubiquitination. The reversal effect of sh‐CAMK1 on sh‐SMURF1‐mediated cell functions suggested that SMURF1 decreased CAMK1 expression to enhance renal cell mitochondrial fission and apoptosis, confirming the anti‐mitochondrial fission and anti‐apoptosis roles of CAMK1, which was consistent with our previous research.^18^ Further animal studies revealed that CAMK1 silencing also reversed the alleviating effect of sh‐NSUN4 on STZ‐induced diabetic mice renal injury. Unlike our previous finding that IGF2BP3 stabilized CAMK1 mRNA via m6A modification, the present study reveals an entirely distinct epigenetic mechanism wherein NSUN4‐catalyzed m5C modification promotes SMURF1 expression, which in turn degrades CAMK1 protein via ubiquitination.

Based on our findings, we believe that NSUN4 likely catalyzed m5C in SMURF1 mRNA, which recruited m5C readers (ALYREF) to stabilize the transcript or promote its translation, thereby increasing SMURF1 protein levels. As an E3 ligase, SMURF1 might directly ubiquitinate CAMK1, targeting it for proteasomal degradation. Next, loss of CAMK1 increased mitochondrial fission markers, leading to excessive mitochondrial fragmentation. Finally, fragmented mitochondria regulated MMP depolarization and triggered apoptosis in renal cells, thus accelerating renal injury in DN.

However, there are some limitations in this study. First, the specific sites of SMURF1 mRNA modification by NSUN4/ALYREF via m5C remain unknown. Second, the included clinical sample size was small, which limited statistical power. Third, pathological mitochondrial fission is usually not an isolated event. It is often accompanied by defects in mitophagy clearance mechanisms such as the PINK1/Parkin pathway, and reduced new mitochondrial generation (PGC‐1α‐dominated biogenesis) constitutes a total breakdown of mitochondrial quality control.^40^, ^41^ Although this study focused on the direct regulation of fission/fusion balance by the NSUN4/SMURF1/CAMK1 axis, whether this axis also simultaneously influences PINK1/Parkin mediated mitophagy or PGC‐1α‐driven biogenesis is an important direction to explore in the future. In our future work, elucidating this aspect will reveal more completely the central role of the NSUN4/SMURF1/CAMK1 axis in mitochondrial dysfunction in DN. Fourth, after the establishment of the STZ‐induced DN model, the body weight of the mice was reduced, and the blood glucose level was increased, showing typical diabetic symptoms.^42^ However, NSUN4‐cKO increased body weight and decreased blood glucose level. This is mainly due to insulin deficiency and insulin resistance caused by long‐term hyperglycaemia after the occurrence of DN, which leads to the inability to use glucose for energy and to break down protein and fat, and then induces weight loss, which is consistent with previous studies.^18^, ^43^, ^44^ However, knockout of NSUN4, as a protective mechanism under DN pathological conditions, was demonstrated in this study to be achieved by inhibiting mitochondrial fission in renal tubular epithelial cells and improving renal function damage. Importantly, it is not clear whether this effect is the result of a direct action on the kidney or an indirect result of correcting the metabolic abnormalities that lead to DN. Finally, IF staining showed that NSUN4 was mainly expressed in the cytoplasmic region, which was not inconsistent with the reported mitochondrial localization. Because mitochondria themselves are located in the cytoplasm, it is difficult to accurately distinguish mitochondria from the surrounding cytoplasm under conventional IF microscopy. Future studies should use more sophisticated fractionation techniques combined with immunoelectron microscopy or live cell imaging to clarify the precise subcellular distribution of NSUN4 under different stimulation conditions.

## 5 CONCLUSIONS

**FIGURE 9:**
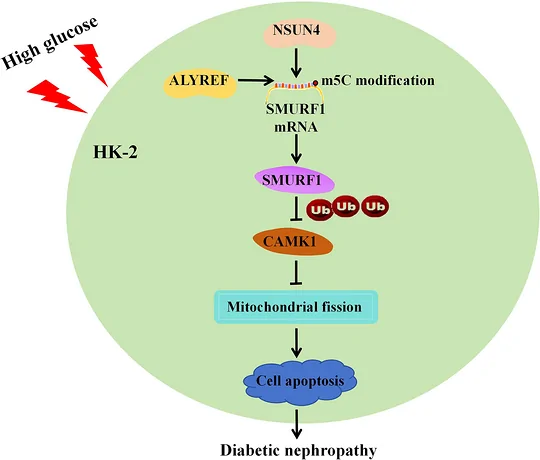
Mechanistic diagram of the present study. NSUN4 interacts with m5C reader ALYREF to regulate the SMURF1/CAMK1 axis, thus promoting HG‐induced HK‐2 cell apoptosis by enhancing mitochondrial fission.

Collectively, our study revealed that NSUN4 promoted mitochondrial fission to accelerate DN‐induced renal injury and HG‐induced renal cell apoptosis, which might be achieved by promoting the m5C modification of SMURF1 to accelerate the ubiquitination and degradation of CAMK1 (Figure 9). The proposal of the NSUN4/SMURF1/CAMK1 axis provides new evidence that mitochondrial dynamics mediates DN progression, and offers a potential molecular target for DN treatment.

## AUTHOR CONTRIBUTIONS

Danyi Yang designed the study and wrote the manuscript. Yinyin Chen and Yang Gao carried out the experimental part of the study. Guoyong Liu supervised the experimental part of the study. Hao Li and Liyu He performed the data analysis. All authors reviewed and approved the final manuscript.

## ETHICS STATEMENT

Each participant signed written informed consent. This study was approved by the Ethics Committee of the Second Xiangya Hospital of Central South University (Approved number: Z0277).

All animal experiment protocols were carried out in accordance with the procedures approved by the Ethics Committee of Second Xiangya Hospital of Central South University (Approved number: 20230326).

## CONFLICT OF INTEREST STATEMENT

The authors declare no conflicts of interest.

## Supporting information




**Figure S1: The expression of NSUN4, SMURF1 and CAMK1 in db/m and db/db mice**. (A, B) NSUN4 mRNA and protein levels were tested using qRT‐PCR and WB in the db/m and db/db groups. (C, D) SMURF1 mRNA and protein levels were checked by qRT‐PCR and WB in each group. (E) CAMK1 protein expression was examined by WB in each group. The data are presented as the mean ± SD. (A–E) Student's *t*‐test. *n* = 8, **p < .01.

**Figure S2: The expression location of NSUN4 in cells**. IF staining was used to analyze the location of NSUN4 in HK‐2 cells under HG or NG conditions.

**Figure 3S: Effect of NSUN4‐cKO on mitochondrial core function parameters in STZ‐induced DN mice**. (A) MitoSOX Red was used to measure mitochondrial ROS levels in the NSUN4‐WT, NSUN4‐cKO, STZ+NSUN4‐WT, and STZ+NSUN4‐cKO groups. (B) ATP level was checked using an ATP assay kit in each group. (C) Quantitative analysis of the aspect ratio of mitochondria in each group. The data are presented as the mean ± SD. (A–C) One‐way ANOVA with Tukey's post hoc test. *n* = 8, **p < .01.

**Figure S4: Effects of sh‐NSUN4 and sh‐CAMK1 on mitochondrial core function parameters in STZ‐induced DN mice**. (A) MitoSOX Red was used to measure mitochondrial ROS levels in control, STZ, STZ+sh‐NSUN4, and STZ+sh‐NSUN4+sh‐CAMK1 groups. (B) ATP levels were checked using an ATP assay kit in each group. (C) Quantitative analysis of the aspect ratio of mitochondria in each group. The data are presented as the mean ± SD. (A–C) One‐way ANOVA with Tukey's post hoc test. *n* = 8, **p < .01.

## Data Availability

The datasets generated and analysed during the current study are available from the corresponding author upon reasonable request.
